# Decoupling of scandium and rare earth elements in organic (nano)particle-rich boreal rivers draining the Fennoscandian Shield

**DOI:** 10.1038/s41598-023-36195-0

**Published:** 2023-06-26

**Authors:** Franziska Klimpel, Michael Bau

**Affiliations:** 1 CritMET-Critical Metals for Enabling Technologies, School of Science, Constructor University, Campus Ring 1, 28219 Bremen, Germany; 2 Present Address: Bærum Kommune, Sandvika, Norway

**Keywords:** Element cycles, Environmental chemistry

## Abstract

Only few datasets on scandium (Sc) and rare earths and yttrium (REY) in rivers are available and the behaviour of Sc in the hydrosphere is poorly understood. We determined Sc and REY concentrations in the dissolved fraction of twelve boreal rivers in Sweden, which show low conductivity, circumneutral pH and elevated dissolved organic carbon (DOC). Scandium concentrations vary between 189 and 1170 pmol/l and are at the high end of the range reported for rivers worldwide. Unusually high Sc concentrations in the Dalsälven and Västerdalälven could be tracked to the Vanån, a tributary to the headwaters of the latter. Increasing Sc with increasing DOC and Yb concentrations suggest that organic ligands play a major role in the distribution of Sc. The REY_SN_ patterns are similar for all rivers (except the Västerdalälven) and are slightly light REY-depleted with negative Ce and Eu anomalies, and positive Y anomalies. These patterns appear to be a general feature of freshwater draining the Fennoscandian Shield into the Baltic Sea for at least the past 28 years. Our results clearly demonstrate that Sc and REY are fractionated in river waters relative to their crustal source and that they should not be discussed jointly as “REE”.

## Introduction

Together with yttrium (Y, atomic number 39) and the lanthanoids (REE: La to Lu, atomic numbers 57–81), scandium (Sc, atomic number 21) is officially classified as a rare earth element by the International Union of Pure and Applied Chemistry^1^ (IUPAC). However, from igneous systems it is known that compared to Y and the REE (rare earths + yttrium: REY), Sc is characterised by very different geochemical behaviour due to, for example, the profound differences between their ionic radii and hence mineral/melt partition coefficients^2,3^.

Scandium is one of the metals used in enabling technologies such as fuel cell/hydrogen technology and Al–Sc alloys used in light-weight construction^4,5^, that are in short supply but important to economies worldwide. Hence, Sc belongs to the group of *critical* raw materials defined by, for example, the European Commission^6,7^ and the U.S. Geological Survey^8^. As a long-term secure source is currently not available, it is not widely used in industry yet, but this is expected to change with increasing industrial demand and the development of new Sc resources. However, an increase in Sc production and industrial use will almost inevitably lead to an increased release of Sc into the environment. To date, there exist only very few studies on the distribution and behavior of Sc in natural surface waters. The behavior of Sc in the hydrosphere, therefore, is currently only poorly understood. Hence, we here provide the first coherent data set on Sc and REY, which focuses on rivers that besides the presence of hydropower dams are (chemically) pristine.

It appears that in general, Sc concentrations in freshwater are low and vary over about two orders of magnitude in the range of 40–3559 pmol/l^9–12^. Scandium is a particle-reactive element and forms strong bonds with organic ligands^9^ and with OH^−^ and F^−^^13^^,^^14^; these complexes usually dominate Sc speciation in natural waters.

In contrast to Sc, the distribution and behavior of the REY in rivers are reasonably well understood. In general, the dissolved REY (defined as the REY in 0.2 µm-filtered water) show in boreal rivers a relatively flat shale-normalized REY pattern (REY_SN_; “SN” indicating normalization to a post-Archean shale such as the Post-Archean Australian Shale (PAAS^15^) or the European Shale (EUS^16^), but with negative Ce anomalies. Some Swedish rivers are also known to show negative Eu anomalies (e.g.,^17–19^). As particle-reactive elements, the dissolved REY are in rivers and lakes mainly associated with nanoparticles and colloids (NPCs; e.g.,^17,20–24^). Another characteristic of boreal rivers is a strong seasonal variation due to snow melt in spring, when the discharge rates are highest (e.g.,^17,25,26^). Throughout the year, the trace elements are partially leached from the upper soil layer(s), percolate downward and re-precipitate deeper in the soil profile. During the snow melt, the water level and flow rate of the rivers strongly increase, causing wash out of the upper soil layers with their NPCs and associated trace elements into the rivers^17^.

In this study we present and discuss coherent Sc and REY data for 12 boreal rivers in Sweden, which are rich in organic NPCs and which drain the Fennoscandian Shield into the Baltic Sea. Additionally, we present REY data for lakes in Sweden as well as Sc and REY data for rivers and one lake in Germany and one river in France to put the results of this study into a broader context. This study significantly expands the limited data available on Sc in river waters, contributing to a better understanding of the geochemical behavior of Sc in the environment.

## Results

**Figure 1:**
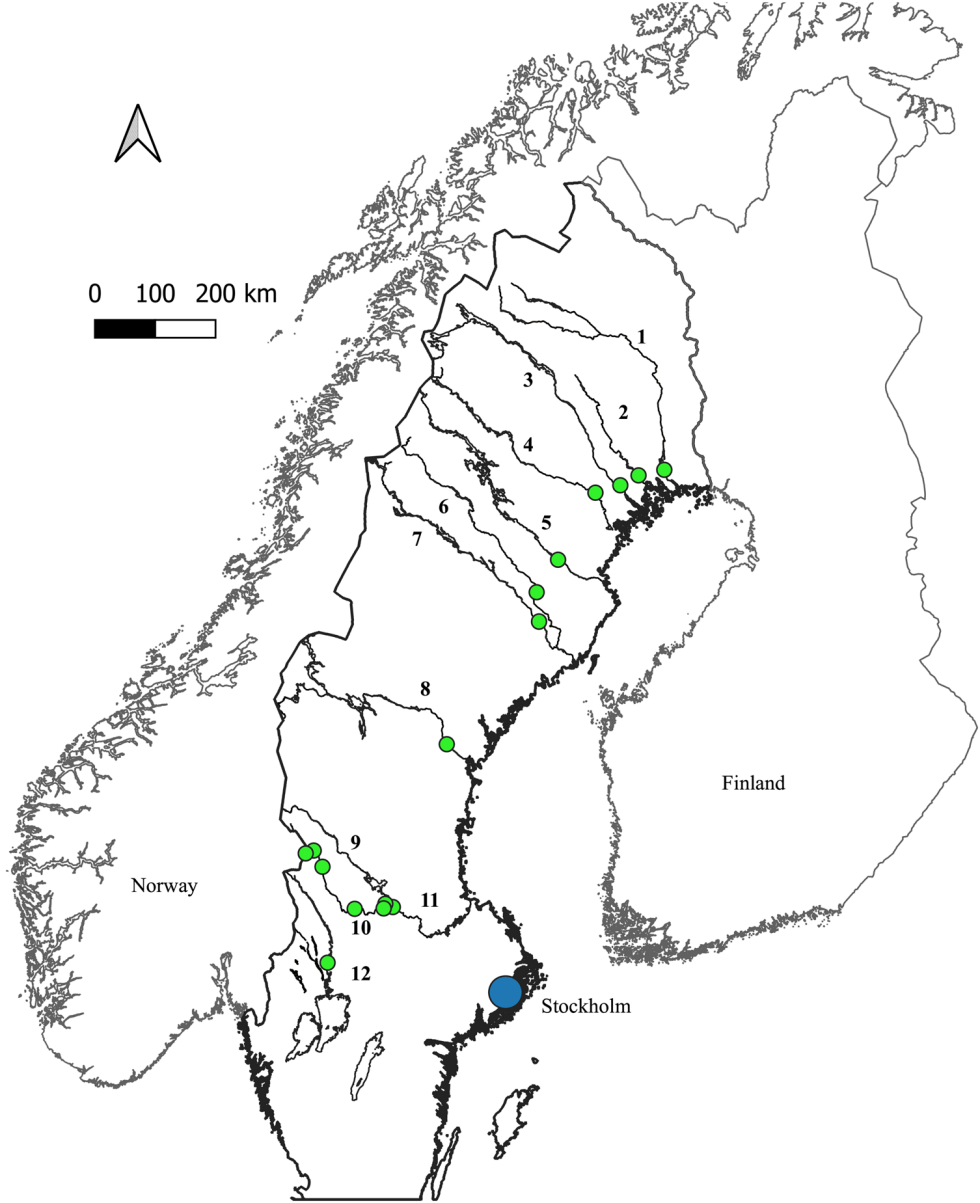
Map showing the sampling spots in Central and Northern Sweden in 2019 and 2022. The precise sampling locations can be found in the supplementary material. (1, Kalixälven; 2, Råneälven; 3, Luleälven; 4, Piteälven; 5, Skellefteälven; 6, Vindelälven; 7, Umeälven; 8, Indalsälven; 9, Österdalälven; 10, Västerdalälven; 11, Dalälven; 12, Klarälven).

The summary of the data of trace elements other than REY and Sc as well as the detailed data tables can be found in the supplementary material. Figure 1 shows the sampling spots in Central and Northern Sweden in 2019 and 2022.

### Swedish rivers in May 2019

#### Conductivity, pH and DOC

All rivers are characterized by rather low conductivity of < 60 µS/cm, the pH values are slightly acidic to neutral (6.04–7.11), and the DOC concentrations vary between a minimum concentration of 5.50 mg/l in the Vindelälven to a maximum of 10.3 mg/l in the Kalixälven.

#### Scandium and REY

**Figure 2:**
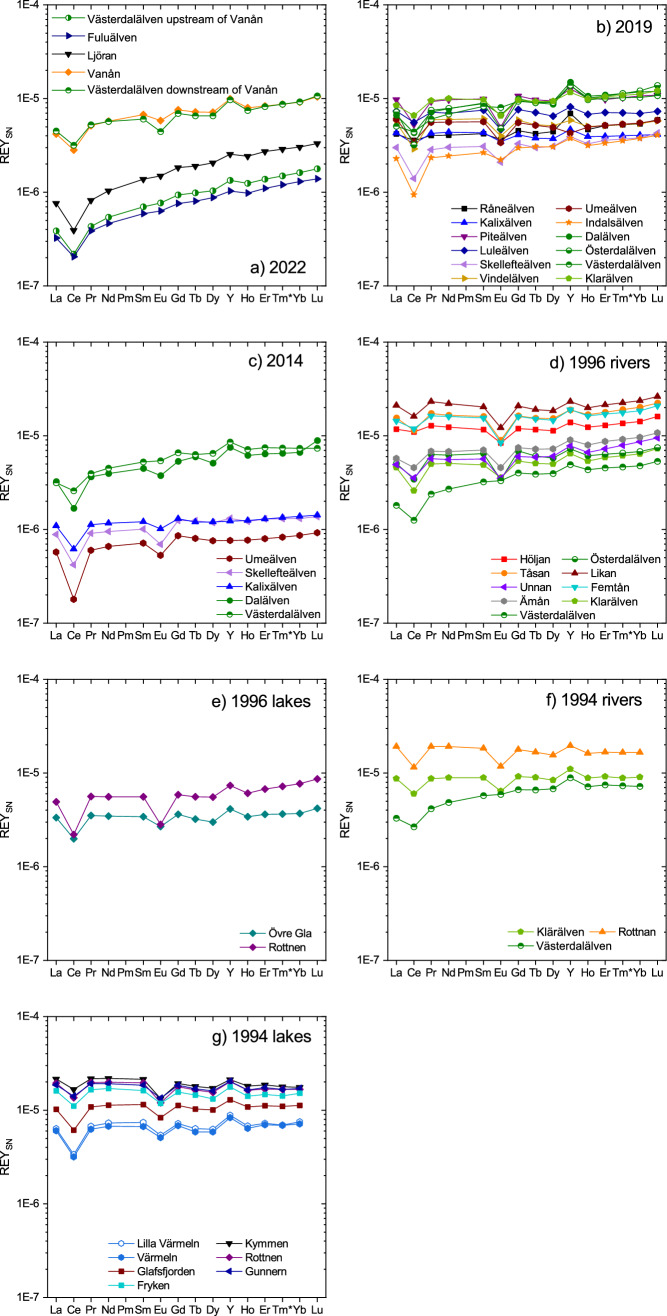
REY_SN_ patterns of the dissolved REY (< 0.2 µm-filtered) for rivers sampled in Central and North Sweden in July 2022 (**a**), May 2019 (**b**) and August 2014 (**c**), and for rivers sampled in September 1996 (**d**) and June 1994 (**f**) and lakes sampled in September 1996 (**e**) and June 1994 (**g**) in Central Sweden.

Scandium concentrations in the 0.2 µm-filtered river waters range from 374 pmol/l in the Skellefteälven to 710 pmol/l in the Klarälven. The eye-catching exception is the Västerdalälven with its extraordinarily high Sc concentration of 1170 pmol/l. Total REY (ΣREY) concentrations are between 4.09 nmol/l (Indalsälven) and 16.4 nmol/l (Klarälven); the Västerdalälven does not show unusually high REY concentrations (ΣREY: 13.5 nmol/l). The Kalixälven, Piteälven, Luleälven, Vindelälven and Umeälven show flat REY_SN_ patterns with negligible fractionation between the LREY, MREY and HREY (La_SN_/Yb_SN_: 0.92–1.09; La_SN_/Tb_SN_: 0.91–1.14; Tb_SN_/Yb_SN_: 0.91–1.02). All other rivers show an enrichment of HREY relative to LREY (La_SN_/Yb_SN_: 0.74–0.87) and are depleted in LREY relative to MREY (La_SN_/Tb_SN_: 0.56–0.75). The Råneälven, Skellefteälven, Indalsälven, Dalälven, Österdalälven, Västerdalälven and Klarälven all show a slight depletion of MREY relative to the HREY (Tb_SN_/Yb_SN_: 0.74–0.87) (Fig. 2b).

The REY_SN_ patterns of all rivers studied show a distinct negative Ce_SN_ anomaly which is most pronounced in the Indalsälven (Ce_SN_/Ce_SN_*: 0.42) and least pronounced in the Råneälven (Ce_SN_/Ce_SN_*: 0.91). Moreover, all rivers (except for the Västerdalälven), show a negative Eu_SN_ anomaly, which is most distinct in the Piteälven (Eu_SN_/Eu_SN_*: 0.482) and least pronounced in the Råneälven (Eu_SN_/Eu_SN_*: 0.84); the Västerdalälven does not show an analytically significant Eu_SN_ anomaly (Eu_SN_/Eu_SN_*: 0.91) (Fig. 2b).

The geochemical twins Y and Ho are slightly fractionated, resulting in positive Y_SN_ anomalies in all rivers and super-chondritic Y/Ho ratios between 31.9 and 40.6.

### Västerdalälven and its tributaries in July 2022

#### Conductivity, pH and DOC

In 2022, the Västerdalälven and its tributaries showed low conductivities (< 35 µS/cm) and circumneutral pH values (6.70–7.67). The DOC concentrations in the Västerdalälven ranged from 2.71 mg/l in the upper part to 8.73 mg/l in the sample furthest downstream. The tributaries show DOC concentrations between 3.10 and 9.79 mg/l for the Ljöran and Vanån, respectively.

#### Scandium and REY

The concentrations of Sc and ΣREY in the Västerdalälven increase strongly from 261 pmol/l and 1.17 nmol/l, respectively, downstream from its confluence with the Fuluälven to 943 pmol/l and 10.2 nmol/l, respectively, downstream of its confluence with the Vanån. In the other tributaries, the Sc concentrations vary between 189 pmol/l in the Fuluälven and 942 pmol/l in the Vanån, and the ΣREY concentration ranges from 0.92 pmol/l in the Fuluälven to 10.1 pmol/l in the Vanån. All rivers show REY_SN_ patterns with enrichments of MREY and HREY over LREY (La_SN_/Tb_SN_: 0.61–0.79; La_SN_/Yb_SN_: 0.39–0.69) and of HREY over MREY (Tb_SN_/Yb_SN_: 0.24–0.49) (Fig. 2a). Furthermore, the REY_SN_ patterns of all rivers show a pronounced negative Ce_SN_ anomaly (Ce_SN_/Ce_SN_*: 0.50–0.59). While the most upstream Västerdalälven sample as well as the samples from Fuluälven and Ljöran show no Eu_SN_ anomaly (Eu_SN_/Eu_SN_*: 0.94–0.95), the Vanån and the Västerdalälven downstream from the Vanån display distinct negative Eu_SN_ anomalies (Eu_SN_/Eu_SN_*: 0.82 and 0.69, respectively). All REY_SN_ patterns yield a positive Y_SN_ anomaly (i.e. super-chondritic Y/Ho ratios between 28.6 and 35.5), indicating slight fractionation of the geochemical twins Y and Ho (Fig. 2a).

#### Swedish rivers and lakes in June 1994, September 1996 and in August 2014

In 2014, the rivers Västerdalälven, Dalälven, Umeälven, Skellefteälven and Kalixälven were sampled and analyzed for their trace metal content including REY. They show ΣREY concentrations between 0.95 nmol/l in the Umeälven and 8.47 nmol/l in the Västerdalälven. Their REY_SN_ patterns (Fig. 2c) are all slightly enriched in HREY over LREY (La_SN_/Yb_SN_: 0.43–0.79) and in MREY over LREY (La_SN_/Tb_SN_: 0.59–0.72), except for the Kalixälven which shows no significant fractionation between MREY and LREY (La_SN_/Tb_SN_: 0.91). There is only a slight or no enrichment of HREY compared to MREY (Tb_SN_/Yb_SN_: 0.85–0.94). All samples show negative Ce_SN_ anomalies (Ce_SN_/Ce_SN_*: 0.29–0.67) and (except for the Västerdalälven) negative Eu_SN_ anomalies (Eu_SN_/Eu_SN_*: 0.62–0.81; Västerdalälven: 0.92). The Y/Ho ratio varies between 26.9 and 33.2 (Fig. 2c).

In 1996, several rivers and lakes in Värmland and Dalarna in Central Sweden were sampled (including the Klarälven, Österdalälven and Västerdalälven) and analyzed for their REY content. The rivers show ΣREY concentrations between 4.81 nmol/l in the Västerdalälven and 36.9 nmol/l in the Likan. All rivers show HREY enrichment compared to LREY (La_SN_/Yb_SN_: 0.38 to 0.89) (Fig. 2d). While the samples from Österdalälven, Unnan, Ämån and Västerdalälven show an enrichment of MREY over LREY (La_SN_/Tb_SN_: 0.46–0.88), the remaining rivers do not show such fractionation (La_SN_/Tb_SN_: 0.90–1.10). Furthermore, there is a slight or no enrichment of HREY over MREY (Tb_SN_/Yb_SN_: 0.68–0.90). The Y/Ho ratios range from 30.6 to 32.9. All samples display negative Ce_SN_ anomalies (Ce_SN_/Ce_SN_*: 0.40–0.84) and (except for the Västerdalälven) negative Eu_SN_ anomalies (Eu_SN_/Eu_SN_*: 0.49–0.76; Västerdalälven: 0.92) (Fig. 2d). The two lake samples show ΣREY concentrations of 5.67 and 8.68 nmol/l. While the Övre Gla shows no fractionation between LREY, MREY and HREY, the Rottnen shows fractionation between LREY and HREY (La_SN_/Yb_SN_: 0.64) and slight fractionation between LREY and MREY (La_SN_/Tb_SN_: 0.88). Both lakes display negative Ce_SN_ anomalies (Ce_SN_/Ce_SN_*: 0.40 and 0.56) as well as negative Eu_SN_ anomalies (Eu_SN_/Eu_SN_*: 0.49 and 0.76) (Fig. 2e).

In 1994, several lakes in Värmland, Central Sweden, as well as the rivers Västerdalälven, Rottnan and Klarälven were sampled and analyzed for their REY content. The rivers have ΣREY concentrations between 8.82 and 30.3 nmol/l. While the Västerdalälven shows a slight depletion of LREY compared to MREY and HREY (La_SN_/Yb_SN_: 0.46; La_SN_/Tb_SN_: 0.50), the Rottnan shows a slight enrichment of LREY compared to MREY and HREY (La_SN_/Yb_SN_: 0.1.16; La_SN_/Tb_SN_: 1.15) and the Klarälven shows no fractionation between LREY, MREY and HREY (La_SN_/Yb_SN_: 0.97; La_SN_/Tb_SN_: 0.97). The rivers show no fractionation between MREY and HREY (Tb_SN_/Yb_SN_: 0.92–1.01) and all rivers have a negative Ce_SN_ anomaly (Ce_SN_/Ce_SN_*: 0.58–0.67) and, except the Västerdalälven, a negative Eu_SN_ anomaly (Eu_SN_/Eu_SN_*: 0.65 and 0.71; Västerdalälven: 0.96). The Y/Ho ratios vary between 32.9 and 33.8 (Fig. 2f). The lakes have ΣREY concentrations between 10.9 nmol/l for Lake Värmeln and 36.3 nmol/l for Lake Kymmen. All lakes show rather flat REY_SN_ patterns with only slight fractionation between LREY, MREY and HREY (La_SN_/Yb_SN_: 0.85–1.23; La_SN_/Tb_SN_: 0.99 to 1.20; Tb_SN_/Yb_SN_: 0.82–1.02). All samples have a negative Ce_SN_ anomaly (Ce_SN_/Ce_SN_*: 0.47–0.75) and a negative Eu_SN_ anomaly (Eu_SN_/Eu_SN_*: 0.64–0.75). The Y/Ho ratio varies between 32.1 for Lake Kymmen and 35.5 for Lake Värmeln (Fig. 2g).

#### Filter residues May 2019 and July 2022

**Figure 3:**
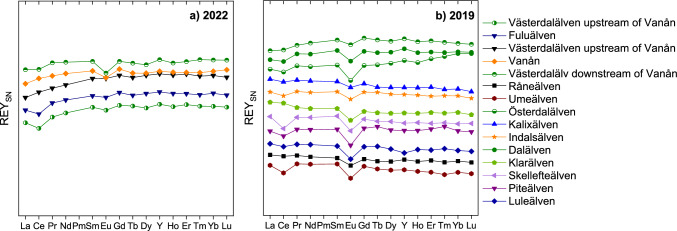
REY_SN_ patterns of the filter residues, i.e. of the suspended particles, of river water samples taken in 2022 (**a**) and 2019 (**b**). Note the absence of units on the y-axis as accurate concentrations could not be determined as explained in the Results section. Arbitrary units are used to facilitate the comparison of individual patterns.

The filter residues represent the suspended particle load of the rivers. As the weight of the filter residues was very low and could hence not be determined with sufficient accuracy, we do not give concentration data, but only the REY_SN_ *patterns* (i.e. relative REY abundances) are presented here. All REY_SN_ patterns are rather flat, resembling those of average upper continental crust (Fig. 3), except for Ce_SN_ and Eu_SN_ anomalies.

In 2019, the REY_SN_ pattern of the filter residue of the Piteälven shows no fractionation between LREY, MREY and HREY, while those of the Österdalälven, theVästerdalälven and the Dalälven are depleted in LREY compared to MREY and HREY (La_SN_/Yb_SN_: 0.60–0.79; La_SN_/Tb_SN_: 0.67–0.89); no fractionation is observed between MREY and HREY. The remaining samples show a slight enrichment of HREY compared to LREY (La_SN_/Tb_SN_: 1.16–1.39). The Y/Ho ratios are close to chondritic and vary between 24.5 and 30.9 for the Luleälven and the Dalälven respectively. The filter residue of the Klarälven shows a slightly positive Ce_SN_ anomaly (Ce_SN_/Ce_SN_*: 1.19), while the filter residues of the Råneälven, Kalixälven, Indalsälven and Luleälven show no Ce_SN_ anomaly (Ce_SN_/Ce_SN_*: 0.90–0.98). The remaining samples show negative Ce_SN_ (Ce_SN_/Ce_SN_*:0.72–0.88) and negative Eu_SN_ anomalies (Eu_SN_/Eu_SN_*: 0.59–0.83) (Fig. 3b).

All filter residues of the 2022 samples show REY_SN_ patterns with an enrichment of LREY over MREY and HREY (La_SN_/Yb_SN_: 0.49–0.73; La_SN_/Tb_SN_: 0.53–0.81) and no fractionation between MREY and HREY (Tb_SN_/Yb_SN_: 0.90–0.94). Furthermore, those of the Västerdalälven and Fuluälven show negative Ce_SN_ anomalies (Ce_SN_/Ce_SN_*: 0.73–0.82), while the samples from the rivers Vanån and Ljöran show no Ce_SN_ anomaly (Ce_SN_/Ce_SN_*: 0.93 and 0.97, respectively). While the filter residues from the Fuluälven and Ljöran rivers have no Eu_SN_ anomaly (Eu_SN_/Eu_SN_*: 0.90 and 0.95, respectively) and the Västerdaläven after those two merge only has a slightly negative one (Eu_SN_/Eu_SN_*: 0.88), the Vanån and the Västerdalälven after it is joined by the Vanån show a negative Eu_SN_ anomaly (Eu_SN_/Eu_SN_*: 0.78 and 0.68, respectively) (Fig. 3a). All Y/Ho ratios are close to chondritic and vary only between 28.2 and 30.4.

### Additional data

As there is only limited Sc data available in literature, we included data for Lake Bullen in north-western Germany as well as data for the rivers Achter, Murg, Ill and Rhine in the discussion. They have Sc and Yb concentrations between 68.1 and 13.5 pmol/l, respectively, for the Rhine River and 414 pmol/l and 83.7 pmol/l, respectively, for the Murg River. The DOC content varies between 1.55 mg/l in the Ill and 5.33 mg/l in Lake Bullen.

## Discussion

### Rare earth elements and yttrium

The REY_SN_ patterns of all river and lake waters sampled in 1994, 1996, 2014 and 2019 show common features such as relatively flat REY_SN_ patterns, negative Ce_SN_ anomalies, negative Eu_SN_ anomalies (except for the Västerdalälven), and positive Y_SN_ anomalies (i.e. decoupling of the geochemical twins Y and Ho). The lakes mostly inherit their geochemical composition from the rivers feeding them, which explains the similarities in the REY_SN_ patterns. The ubiquity and longevity of this REY signature and the fact that it is independent of the exact sampling month (June 1994, September 1996, August 2014, May 2019) suggests that it is characteristic of surface waters in Central and Northern Sweden, and likely in all of the Fennoscandian Shield.

The REY_SN_ patterns of both the water samples and the filter residues show a negative Eu_SN_ anomaly, suggesting that this feature may be inherited from an Eu-depleted local bedrock.

In contrast to the Eu_SN_ anomaly, the filter residues do not show any Y_SN_ anomaly, i.e. there is no decoupling of Y and Ho, and hence the positive Y_SN_ anomaly in the dissolved phase is most likely a result of REY mobilization during weathering^27^ as it shows a slightly lower tendency for surface-complexation with hydroxyl groups than its REE neighbors (e.g.,^28^). Similarly, the negative Ce_SN_ anomaly is most pronounced in the dissolved phase, while the filter residues only show a slightly negative Ce_SN_ anomaly. The Ce_SN_ and Y_SN_ anomalies therefore suggest that the REY-carrying NPCs in the 0.2 µm-filtered waters are mostly authigenic ones, i.e. Fe oxyhydroxides and/or organic NPCs which in Swedish rivers are known to show negative Ce_SN_ anomalies^17^ and to dominate the riverine transport of REE^25^.

There is no evidence of any anthropogenic REY input and even the anthropogenic positive Gd_SN_ anomaly which is by now a common feature of many rivers in Europe and North America (e.g.,^29^), cannot be observed in the major rivers in Central and Northern Sweden, probably due to their high discharge which promotes dilution, their high NPC load that masks the truly dissolved anthropogenic Gd, and their thinly populated catchments where only small amounts of anthropogenic Gd are released into the environment.

The samples taken in August 2014 show lower REY concentrations than those of May 2019. Additionally, their REY_SN_ patterns show a slightly stronger HREY enrichment over LREY and more pronounced negative Ce_SN_ anomalies compared to 2019. This indicates that the water in 2019 contains a larger amount of colloids as this would increase the REY and especially the LREY concentration and therefore leads to a flatter REY_SN_ pattern (e.g.,^20^). In 2019, the samples were taken during the spring flood in May, i.e. during snow melt. During the spring flood, the hydrological pathways in the catchments change, which leads to a strong increase of the surface water component^30^. Due to the snow melt, the O and E horizons of the surrounding podzol soils are washed out, leading to a sharp increase in organic carbon-rich NPCs, Fe, Al and REE^17,25,26^. When comparing the REY concentrations of this study with data from Ingri et al.^17^, it becomes apparent that the REY content of the samples from May 2019 is similar to the REY content observed at maximum spring flood, while the REY content of August 2014 is more similar to concentrations before or after the spring flood. The increase of REY is especially pronounced for LREY as they are more strongly weathered in the upper soil horizons compared to HREY^31^.

### Scandium in boreal organic NPC-rich Swedish rivers compared to rivers elsewhere

**Figure 4:**
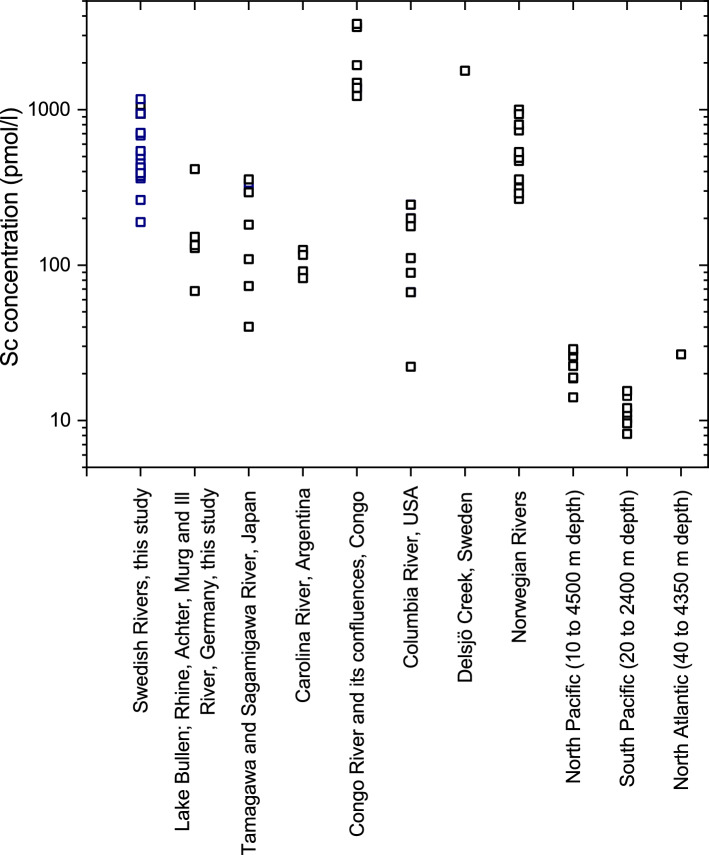
Scandium concentrations (pmol/l) in Swedish rivers and Lake Bullen and the rivers Rhine, Murg, Achter and Ill (Germany; this study) compared to data for the Tamagawa and Sagamigawa rivers, Japan^9^, Carolina River, Argentina^10^, Congo River and its tributaries^12^, Columbia River, USA^11^, Delsjö Creek, Sweden^33^, Norwegian Rivers^34^ together with data for the North and South Pacific and the North Atlantic^35^.

While Sc data for river waters are scarce, it nevertheless appears that the Sc concentrations determined in the Swedish boreal rivers are rather high compared to most of the few data sets available for other rivers in Japan^9^, Argentina^10^, the USA^11^, and Germany (Fig. 4). The Swedish rivers are, however, lower in Sc compared to concentrations observed in the tropical Congo River and its tributaries^12^ which show some of the highest dissolved (in this case 0.45 µm-filtered) trace element concentrations observed in rivers worldwide^12,32^. The Delsjö Creek in Sweden shows Sc concentrations similar to those observed in the Congo River^33^. Unsurprisingly, Sc concentrations in the Swedish rivers fall within the same range as those reported from Norwegian rivers that drain similar bedrocks^34^.

Compared to the range of Sc concentrations reported for seawater^35^ (North Atlantic depth profile: 5.70–30.3 pmol/l; North Pacific depth profile: 0.80–28.8 pmol/l; South Pacific depth profile: 0.30–18.3 pmol/l), the Sc concentrations in river water are substantially higher (Fig. 4), as would be expected for a particle-reactive element the dissolved speciation in seawater of which is dominated by free Sc^3+^ and Sc hydroxide complexes^14^.

### Controls on the Sc distribution in river water

**Figure 5:**
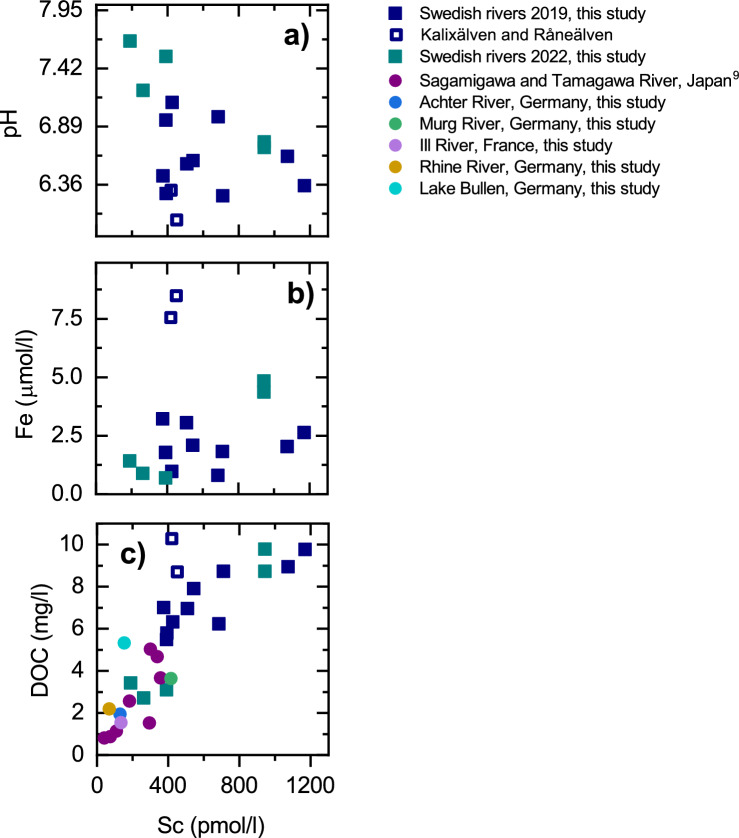
Graphs of Sc concentrations *vs* (**a**) pH value, (**b**) Fe concentrations, and (**c**) DOC concentrations in the boreal Swedish rivers. There is no correlation or trend in (**a**) and (**b**) (r^2^ = 0.130 and 0.003, respectively. Comparison of dissolved Sc concentrations (pmol/l) with dissolved DOC concentrations (mg/l) of the Swedish rivers included in this study, plus two Japanese rivers^9^ and the German rivers Achter, Murg, Ill, Rhine and the Lake Bullen shows that rivers with higher DOC concentrations tend to show elevated Sc concentrations (**c**) (r^2^ = 0.67).

As shown in Fig. 5a and b, there is no clear relationship between Sc concentrations and the pH or the Fe concentration in the boreal Swedish rivers discussed here (r^2^ = 0.13 and 0.003, respectively). However, excluding the Kalixälven and Råneälven in Northern Sweden, which show significantly higher Fe concentrations compared to the other rivers, Sc shows a trend of increasing dissolved concentration with increasing DOC content (r^2^ = 0.73; Fig. 5c). This trend is confirmed when data for DOC-poor rivers (Rhine River and its tributaries Achter, Murg and Ill, Germany and France—this study; Sagamigawa and Tamagawa rivers, Japan^9^) are included (Fig. 5c) as this larger data set also reveals a clear positive relationship between Sc and DOC concentrations (r^2^ = 0.67).

Dahlqvist et al.^26^ observed a peak in Sc concentration in the Kalixälven in Northern Sweden during snow melt. Unfortunately, they did not publish any concentration data, which prevents comparison of their data to our results. However, their observation may suggest that the rather high Sc concentrations observed in the Swedish compared to other rivers may partially be an effect of the seasonal snow melt, as has also been observed for other trace elements such as the REE^26^. Furthermore, Tanizaki et al.^9^ observed increased Sc concentrations during a heavy rain fall event that was accompanied by flooding in the Tamagawa and Sagamigawa rivers in Japan. The Swedish and Japanese data suggest that dissolved Sc concentrations may depend on the abundance of NPCs in a river, similar to what is observed for the REY (e.g.,^21^). The significantly lower Sc concentrations observed in seawater support this conclusion, as NPCs aggregate during seawater-freshwater mixing in the low-salinity part of estuaries and are deposited. This process also removes the particle-reactive elements which are associated with the NPCs and reduces the dissolved flux of these elements into seawater (e.g.^36,37^).

### Relationship between Sc and the REY

Scandium is formally included in the group of rare earth elements by the IUPAC^1^. However, one of the main characteristics of the REE is their coherent behavior in natural systems which is due to their same charge and their similar yet systematically decreasing ionic radii. Yttrium is often discussed together with the REE as it is the geochemical twin of Ho, showing the same charge and very similar ionic radius (e.g.,^38^). This results in smooth normalized REY_SN_ patterns for most natural materials. However, from igneous systems it is known that Sc behaves very different from the REY as it partitions into other minerals than the REY do because of its much smaller ionic radius^2,3^. As a result, Sc preferentially partitions into Fe- and/or Mg-rich mafic minerals while the REY are strongly incompatible in silicate magmas and remain in the melt. The overall result is a systematic increase of REY/Sc ratios during igneous differentiation, i.e. low REY/Sc ratios in mafic and high REY/Sc ratios in felsic igneous rocks. These REY/Sc ratios are transferred to clastic sediments and sedimentary rocks and the La/Sc ratio, for example, is thus used as a geochemical proxy (similar to the Th/Sc ratio) for the chemical evolution of a sediment source area^15^. Hence, referring to Sc as a rare earth element makes little sense for igneous systems and epiclastic sediments and sedimentary rocks.

**Figure 6:**
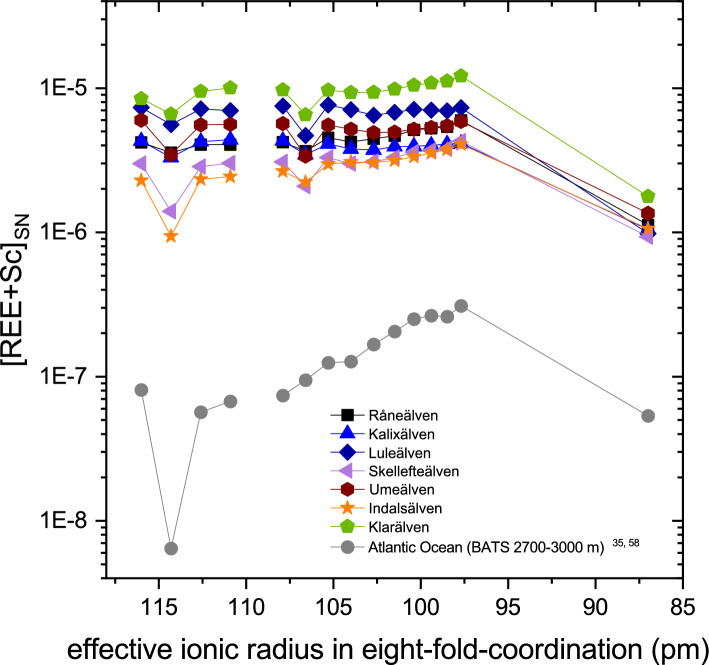
REE_SN_ pattern with REE positioned according to their ionic radius along the x-axis and including Sc. In addition to data from this study, REE and Sc data from the Atlantic Ocean is included^35,58^ (Bermuda Atlantic Time Series (BATS) site; 2700–3000 m water depth).

Compared to igneous systems, only very little is known about the Sc-REY systematics of aqueous systems such as natural waters. In Fig. 6, the shale-normalized REE and Sc concentrations of selected Swedish rivers and of examples of Atlantic and Pacific seawater are plotted against their ionic radius in eight-fold coordination. These “REESc_SN_”-patterns reveal that Sc does not fit at all into a smooth normalized REE_SN_ pattern. Hence, as in igneous and epiclastic sedimentary rocks, Sc should be presented and discussed separately from the REE in geochemical studies of aqueous fluids and their precipitates (including chemical sediments). Moreover, comparison of Sc and REY data for the river waters (i.e. the 0.2 µm-filtrates) and their respective suspended particles (i.e. the filter residues) reveals significant decoupling of Sc from REY during weathering as the Sc/Yb ratios of the river waters are 1.6–5 times higher than the Sc/Yb ratio in the suspended particles.

### Controls on the behavior of Sc

**Figure 7:**
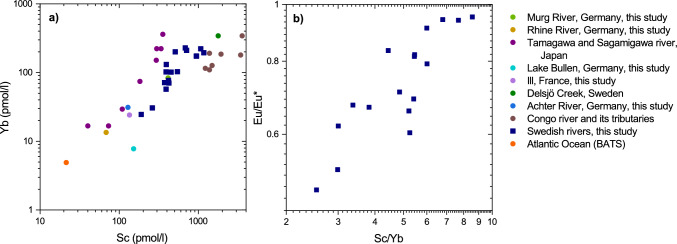
Graphs of Sc concentrations vs Yb concentrations show a positive relationship also when including data for two Japanese rivers^9^, Delsjö Creek^33^, Congo River and its tributaries^12^, the Atlantic Ocean^35,58^ and the rivers Achter, Murg, Ill, and Rhine, and the Lake Bullen (this study) (**a**). Additionally, a positive trend between Sc/Yb and Eu/Eu* can be observed for the Swedish rivers (**b**).

In our dataset, Sc shows a positive relationship with Yb and the HREY (r^2^ = 0.64), despite the strong decoupling of REY and Sc observed in the REESc_SN_ patterns (Fig. 6). This relationship holds if the data set is enlarged and published Yb-Sc data are included (Fig. 7a). This may indicate that once they have been mobilized during weathering, there is negligible or only very minor further decoupling between Sc and the REY in river water.

Ytterbium like the other HREY has a high affinity for dissolved organic ligands such as polycarboxylic acids and organic-rich colloids^39–42^. Previous studies of boreal rivers showed that organic-rich colloids are the main carrier phases for Yb and other REY during the snow melt^26^. It is very likely that this is no different for Sc as it shows higher stability constants for organic ligands such as EDTA^43^ compared to Yb. However, further studies are needed to better understand the influence of NPCs on the biogeochemical behavior of Sc. We showed above that there is generally a positive relationship between DOC and Sc concentrations when including literature data, suggesting that organic ligands play an important role in controlling the geochemical behavior of Sc. Tanizaki et al.^9^ also showed a correlation between Sc and DOC, which varied between the different size fractions, suggesting that not all organic ligands referred to as “DOC” bind equally strong to Sc.

Furthermore, the Swedish rivers show a positive relationship between Sc/Yb and Eu/Eu* (r^2^ = 0.73; Fig. 7b). As described above, NPCs dominate the geochemical behavior of Sc and the REY in the dissolved phase, and therefore also control these two ratios. A comparatively higher Sc/Yb ratio could indicate a more mafic origin of these NPCs as Sc on average has higher concentrations in mafic rocks compared to felsic rocks^44^, while Yb (and the other REY) shows higher concentrations in felsic rocks. As the negative Eu_SN_ anomaly likely is inherited from the local bedrock as explained above, a low Sc/Yb ratio and a larger negative Eu_SN_ anomaly could indicate a chemically more evolved catchment lithology.

### Anomalous Sc enrichment in the Västerdalälven and Dalälven

In May 2019, two samples show significantly higher Sc concentration compared to the other samples: the Västerdalälven (which also shows anomalous concentrations for several other trace elements) and the Dalälven. The latter forms as the result of the confluence (and hence mixing of the waters) of the Västerdalälven and the Österdalälven and reflects the anomalously high Sc concentration of the Västerdalälven. This is corroborated by mixing calculations (Table S10). The calculation of mixing ratios between the Västerdalälven and Österdalälven using elements that behave conservatively such as Sr or Ca, suggests that the Österdalälven supplies between 42 and 54% to the bulk geochemical composition the Dalälven, which fits well with the 55% estimated based on flow rates of the respective rivers^45^. Using the REY and Sc concentration of the endmembers Västerdalälven and Österdalälven and assuming that the Österdalälven contributes 54% to the Dalälven yields theoretical REY concentrations that are within 10% of the measured concentrations in the Dalälven. The suggested conservative mixing of REY agrees with the conservative behavior of organic colloids^36^, which are the main carrier of REY in the dissolved phase in the boreal rivers. The theoretical Sc concentration obtained is 18% lower than the measured concentration, possibly indicating slightly non-conservative mixing.

The Västerdalälven, therefore, is the only exception from the general Sc range observed in the samples taken in 2019 from the major Swedish rivers that drain the Fennoscandian Shield into the Baltic Sea. To verify this observation, the upper reaches of the Västerdalälven and its tributaries Fuluälven, Ljöran and Vanån were sampled in July 2022 (note that this was after snow melt). The results show that upstream of the confluence of Vanån and Västerdalälven, the Sc concentration in the Västerdalälven and in its upper tributaries Fuluälven and Ljöran is rather low (< 400 pmol/l). The true exception is the Vanån which has a significantly higher Sc concentration (942 pmol/l). This river also shows significantly higher REY, Fe and DOC concentrations compared to the other tributaries and to the Västerdalälven upstream of the confluence. Further downstream, the chemical composition of the Västerdalälven is dominated by the element input from the Vanån, as revealed by the similarity in DOC, Sc, REY and Fe concentrations as well as the REY_SN_ pattern (Fig. 2a and table S2). The high Sc concentrations and REY content in the Vanån may be explained by a higher amount of organic colloids present in the dissolved phase, as indicated by the elevated DOC content. The upper Västerdalälven as well as the Fuluälven and Ljöran all have low DOC concentrations compared to the range observed in 2019. One reason could be that the 2019 samples were taken during the snow melt in May, and hence showed higher trace element concentrations as well as a higher (nano)particle load^17^. The relatively strong depletion of LREY compared to HREY in 2022 indicate that less nanoparticles and colloids are present in the dissolved phase. Despite the differences in sampling seasons and the differences in the discharge rate (Table S2), the Sc concentrations in the Västerdalälven are similarly high. This indicates that the high Sc concentrations observed in the river in May 2019 compared to the other rivers sampled cannot solely be explained by the snow melting event but is most likely caused by the influence of the Vanån.

## Conclusion

Scandium concentrations in boreal rivers that drain the Fennoscandian Shield in Sweden and which are rich in organic NPCs, range from 189 to 1170 pmol/l. However, high concentrations observed in the Dalälven and Västerdalälven are most likely caused by the Vanån, a tributary to the headwaters of the Västerdalälven. Excluding these samples, the maximum concentration in the Swedish rivers is 710 pmol/l.

The REY distribution in the rivers studied did not change significantly over the past 28 years since the mid-1990s and lakes show a similar REY composition compared to the rivers. Their REY_SN_ patterns are characterized by a slight enrichment of HREY compared to LREY, negative Ce_SN_ and Eu_SN_ anomalies as well as positive Y_SN_ anomalies. The distribution of the REY in the 0.2 µm-filtered waters is mainly controlled by NPCs present in the “dissolved” phase and the concentrations appear to increase during the snow melt due to a higher concentration of NPCs in the rivers.

The Sc concentrations observed correspond well with Sc concentrations of Norwegian rivers but are higher compared to Sc concentrations observed in most other rivers worldwide as well as in seawater. Scandium behavior in the Swedish rivers and lakes seems to be strongly affected by organic NPCs as indicated by the trend of increasing Sc concentrations with increasing concentrations of DOC and Yb (and other REY). Furthermore, Sc is in river and lake waters strongly fractionated from the REY, relative to the upper continental crust (represented by shale) from which these elements are sourced. Thus, despite being officially (and commonly) classified as a rare earth element, Sc should be discussed separately in geochemical (and probably biological) studies. Nevertheless, the covariation between the Sc/Yb ratio and the Eu/Eu* ratio (i.e. the Eu_SN_ anomaly) suggests that Sc-REY relationships may provide information on the overall catchment lithology.

## Methods

The following section gives a detailed description of the sampling, preconcentration and analytical methods of the samples taken in Sweden in 2019 and 2022 as well as the samples from Germany and France. A detailed description of the analytical procedure for the samples from 1996 and 2014 can be found in Bau and Dulski^46^ and Kulaksiz and Bau^29^, respectively.

### Sampling

Samples were taken in acid-cleaned polyethylene bottles from twelve different rivers in Central and Northern Sweden in May 2019 as well as from the Västerdalälven and its tributaries in July 2022 (Fig. 1). Samples from the Lake Bullen as well as from the four rivers in Germany and France were taken in 2020. Conductivity, pH, and temperature were measured directly in the field (with a WTW340i meter). A sample for DOC analysis was taken and filtered on-site with 0.2 µm polyethersulfone filters and acidified with HCl. All other water samples were filtered with 0.2 µm cellulose acetate filters. In total, 2 l of water were taken in 2019, which after filtration were split into two 1000 ml aliquots for pre concentration with Nobias PA-1 W columns (acidified with HCl and HF) and with precleaned ion exchange Sep-Pak C18 cartridges containing ethyl-hexyl phosphate (acidified with HCl), respectively. During sampling in 2022, 1 l of water was taken and later preconcentrated with Nobias PA-1 W columns (acidified with HCl and HF). Before preconcentration, 10 ml H_2_O_2_ of supra-pure quality were added to the samples which were then stored at 70 °C for 5 days to dissolve any organic compounds.

### Preconcentration procedure

Nobias PA-1 columns (Hitachi High Technology) were used in several previous studies to preconcentrate REY from seawater and estuarine water (e.g.,^47–49^). Parker et al.^35^ modified the method to preconcentrate Sc from seawater and this protocol was further adapted for our study. All chemicals used were of supra-pure quality. A 25 ppt Tm spike was added to all samples and a 50 ml aliquot was taken prior to preconcentration to monitor Tm spike recovery. Before preconcentration, a 3.6 M ammonium acetate buffer was prepared, that was further diluted to a 0.05 M ammonium acetate buffer (pH adjusted to 6). The Nobias PA-1 columns were cleaned with 30 ml of 3 M HNO_3_ and 0.002 M HF at a pump speed of 1 ml/min, before 60 ml of 0.05 M ammonium acetate buffer were passed over the column at 2 ml/min. Immediately before preconcentration, 3.6 M ammonium acetate buffer was added to the sample, so that the final concentration in the sample was 0.05 M and the pH was adjusted to between 6.0 and 6.1. The sample was then passed over 2 columns simultaneously, using a Y connector at a pump speed of 2 ml/min. Afterwards, another 60 ml of 0.05 M ammonium acetate buffer was passed over the column before the sample was eluted with 50 ml of the 3 M HNO_3_ and 0.002 M HF acid mix at 1 ml/min. The eluate was evaporated and taken up in 10 ml of 0.05 M HNO_3_, so that the final preconcentration factor was about 100.

The second aliquot was preconcentrated using precleaned C18 cartridges loaded with ethyl-hexyl phosphates, which is a well-established method the detailed protocol of which can be found elsewhere^46,50,51^. This second type of preconcentration was done to control the method reliability of the preconcentration with the Nobias PA-1 column for REY in freshwater as the use of this method for freshwater samples is less well established than its application in seawater analysis. Furthermore, the data from this study are compared to unpublished REY data for samples taken and analyzed in 1994 and 2014, which were preconcentrated by this method. It was, therefore, important to successfully verify that the different preconcentration techniques do not affect the REY data produced.

### Filter residues

The filters were dried at 70 °C and then digested with a mix of supra-pure HCl, HNO_3_ and HF at 200 °C using a PicoTrace DAS acid pressure digestion system. After evaporation to incipient dryness at 200 °C, the samples were redissolved twice with 5 ml HCl and evaporated before they were taken up in 50 ml of 0.5 M HNO_3_ and analyzed. For quality control, 0.05 g of the reference material SCo-1 was weighed in and digested parallel to the filter residues.

### Analysis

A quadrupole Inductively Coupled Plasma Mass Spectrometry (ICP-MS; Perkin Elmer NexION 350xs) coupled with an APEX2 (Elemental Scientific) was used to determine the concentrations of Sc, REY and other trace elements. Furthermore, the samples were also analyzed for additional elements by Inductively Coupled Plasma Optical Emission Spectrometry (ICP-OES; SpectroCiros Vision). While Y was used as internal standard for the ICP-OES analysis, Ru, Re, and Bi were used as internal standard for ICP-MS analysis. For the analyses of water samples, the certified reference material SLRS-6 was used for quality control, while a SCo-1 was used as a reference material for the filter residues. Due to several polyatomic interferences on the mass of ^45^Sc^52^, some samples were additionally analyzed using the kinetic energy dispersion (KED) mode after the preconcentration and matrix separation with the Nobias PA-1 columns.

### Method reliability

De-ionized water (DI) was subjected to the same analytical procedure as the water samples to characterize a method blank. All analyte concentrations were found to be below the lower limit of quantification (LOQ).

Our ICP-OES-determined results for the SLRS-6 standard are in very good agreement (RSD < 8%) to published data^53^; two samples (Klarälven and Kalixälven) that were measured in duplicate, are also in very good agreement (< 8%). Furthermore, the results for the SLRS-6 standard measured with the ICP-MS is also in close agreement (< 10%, except Lu: 15%) with published data for the REY and other trace elements^53^. Scandium is ill-defined in freshwater standards in general and to the best of our knowledge there are currently only three published values for the SLRS-6 (0.207^54^, 0.363^55^ and 7.41 nmol/kg^53^) which show an unrealistically large difference. We emphasize that the Sc concentration determined by our approach (0.397 nmol/kg) is much closer to the result of Sugiyama^55^.

Comparison between the measurement with and without KED mode for selected water samples and for the SLRS-6 standard are in very good agreement (RSD < 5%), suggesting that polyatomic interferences are negligible when analyzing Sc after the preconcentration and matrix separation using Nobias PA-1 columns.

Recovery of the Tm spike was good for all samples with values between 91 and 104% in 2019 (except for Luleälven, which shows a Tm recovery of 86%) and between 99 and 110% in 2022 for the preconcentration with the Nobias PA-1 columns and between 99 and 107% for the preconcentration with the C18 cartridges.

**Figure 8:**
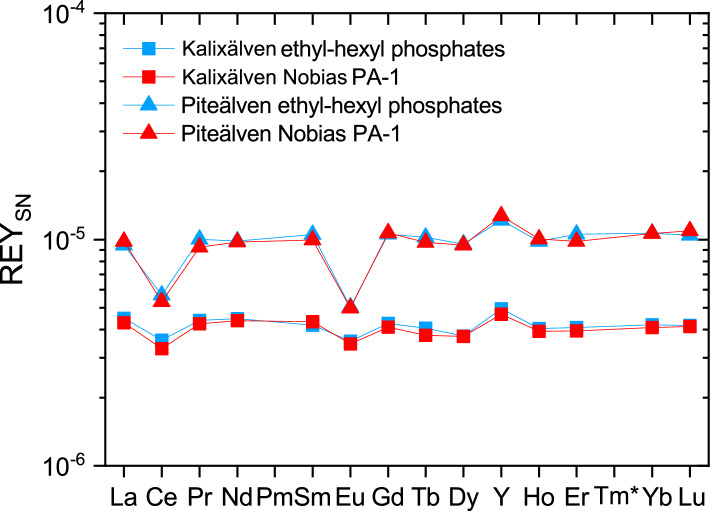
Dissolved REY_SN_ patterns of Kalixälven and Piteälven after preconcentration and matrix separation with Nobias PA-1 columns and C18 cartridges loaded with ethyl-hexyl phosphates, respectively. The REY_SN_ patterns are in very close agreement, demonstrating that both methods are equally well-suited to separate and preconcentrate REY from river water, allowing straightforward comparison between REY data determined for samples from 1994, 2014 and 2019. Note that Sc cannot be reliably preconcentrated using the C18 cartridges filled with ethyl-hexyl phosphates and hence the Nobias PA-1 columns must be used.

The REY concentration obtained using the two different preconcentration methods are in very close agreement with each other (Fig. 8), confirming that the preconcentration procedure with the Nobias PA-1 columns is suitable to preconcentrate all REY together with Sc from river water, and that no analytical bias hampers comparison of the new data for Swedish rivers in 2019 with the yet unpublished data from 1994 and 2014.

### Reporting of data

The REY are subdivided into light REY (LREY: La–Gd) and heavy REY (HREY: Tb–Lu, incl. Y), sometimes reference is also made to the middle REY (MREY: Sm–Dy). All REY data presented here are normalized to European Shale^16^ (subscript “SN”). The Ce_SN_ anomaly was quantified following Eq. (1)^56^:

1 $${\text{Ce/Ce}_{\text{SN}}}^*\;{ = }\;{\text{Ce}_\text{SN}/10}^{{(2{\text{log(Nd}} _\text{SN})- {\text{log(Sm}}_\text{SN}))}}$$

The Eu_SN_ anomaly was calculated using the following equation:

2 $${\text{Eu}_\text{SN}/\text{Eu}_\text{SN}}^* = {\text{Eu}_\text{SN}/10}^{{{(0}{\text{.5log(Sm}}_\text{SN})+ 0{\text{.5log(Gd}}_\text{SN}))}}$$

As Tm was used as a spike, Tm concentrations in the samples could not be determined and have been calculated (Tm*) following Eq. (3)^57^, where [Tm]_EUS_ is the Tm concentration in the European Shale^16^:

3a $${\text{Tm}_\text{SN}}^*\;{ = }\;{\text{Tm}_\text{SN}} \times {10}^{{{(0}{\text{.5log(Er}}_\text{SN})+ 0{\text{.5log(Yb}}_\text{SN}))}}$$

3b $$\left[ {{\text{Tm}}} \right]^* \, = {{\text{ Tm}}_{\text{SN}}} ^* \, \times \, \left[ {{\text{Tm}}} \right]_{{{\text{EUS}}}}$$

## Supplementary Information

Supplementary Information.

## Data Availability

All data generated or analyzed during this study are included in this published article (and its Supplementary Information).
