# Supplementary material for: Decoupling of scandium and rare earth elements in organic (nano)particle-rich boreal rivers draining the Fennoscandian Shield
Source: Sci Rep. 2023 Jun 26;13:10357. doi: 10.1038/s41598-023-36195-0 (PMC10293264; doi:10.1038/s41598-023-36195-0)
Supplement: Supplementary file 1 — Supplementary Information. [file 41598_2023_36195_MOESM1_ESM.pdf]

Decoupling of scandium and rare earth elements in organic (nano)particle-rich boreal rivers draining the Fennoscandian Shield

Franziska Klimpel[1*], Michael Bau[1]

[1] CritMET – Critical Metals for Enabling Technologies, School of Sciences, Constructor University, Campus Ring 1 - 28219 Bremen, Germany

[*]Corresponding author address: fklimpel@constructor.university

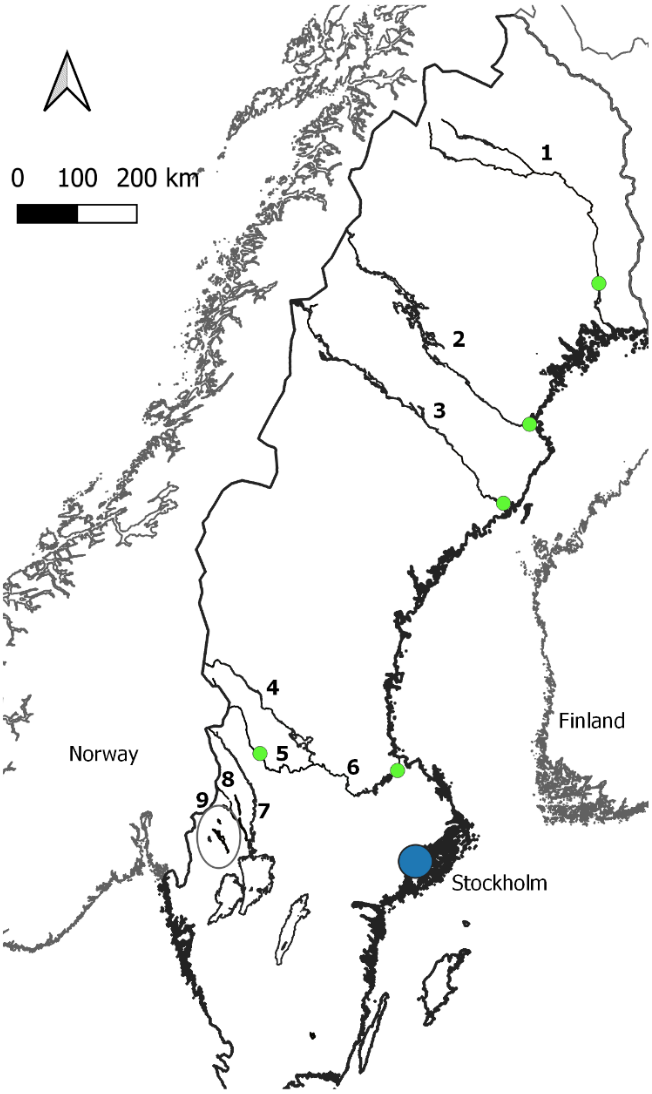

Supplementary Figure S1 Map showing the sampling sport in Sweden in June 1994, September 1996 and August 2014. 1: Kalixälven; 2: Skellefteälven; 3: Umeälven; 4: Österdalälven; 5: Västerdalälven; 6: Dalälven; 7: Klarälven and its tributaries from north to south Höljan, Tåsan, Likan and Femtån; 8: Fryken; 9: the lakes Övre Gla, Glafsfjorden, Kymmen, Gunnern, Rottnen and Värmeln

Supplementary Table S2 REY, Sc and other trace element data for Swedish rivers sampled in 2019 and 2022. The Ce_{SN} anomaly calculations are based on eq. 1, the Eu_{SN} anomaly calculations are based on eq. 2 and Tm* was calculated following eq. 3a and 3b. The flow rate data was taken from Swedish Meteorological and Hydrological Institute (2023)[1].

sample name	Klarälven	Österdalälven	Västerdalälven	Dalälven	Indalsälven
sampling date	22.05.2019	22.05.2019	22.05.2019	22.05.2019	21.05.2019
latitude	59.771213	60.588457	60.524741	60.542845	62.699463
longitude	13.458816	15.070075	15.023544	15.27912	16.783389
flow rate (m^3/s)	184	195	173	355	514
pH	6.26	6.98	6.35	6.62	7.11
conductivity (µS/cm)	20.0	30.0	19.0	25.0	60.0
temperature (°C)	11.1	11.3	11.5	11.3	9.50
DOC (mg/l)	8.73	6.23	9.77	8.95	6.33
µmol/l					
Ca	55.6	93.6	59.6	76.6	255
Fe	1.83	0.813	2.63	2.04	0.972
Na	42.0	58.3	39.5	49.2	53.1
Si	68.0	99.7	81.9	90.4	51.6
nmol/l					
Rb	10.9	12.6	8.93	10.6	9.49
Sr	101	184	104	137	275
Cs	0.0323	0.0678	0.079	0.0711	0.0342
Ba	134	89.8	63.8	77.9	36.4
U	0.214	0.959	0.277	0.629	1.08
pmol/l					
Sc	710	683	1170	1070	425
Y	4170	4950	5000	5350	1350
La	2690	2300	1610	2120	730
Ce	4170	2020	2790	2720	593
Pr	714	566	455	540	175
Nd	2740	2160	1880	2140	667
Sm	472	429	402	435	129
Eu	63.6	46.6	78.3	65.0	21.6
Gd	390	383	378	399	120
Tb	55.3	53.4	53.6	54.5	18.2
Dy	337	314	328	335	110
Ho	69.7	71.8	68.8	74.9	22.3
Er	214	217	207	223	68.4
Tm*	31.5	33.0	29.7	32.9	10.3
Yb	211	229	195	221	70.6
Lu	33.6	38.3	29.9	33.3	11.3
Tm recovery (%)	101	99.2	103	104	92.5
Sum REY (nmol/l)	16.4	13.8	13.5	14.7	4.09
Y/Ho	32.3	37.1	39.2	38.5	32.6
La_{SN}/Yb_{SN}	0.905	0.803	0.561	0.723	0.745
La_{SN}/Tb_{SN}	0.832	0.741	0.874	0.781	0.819
Tb_{SN}/Yb_{SN}	0.753	0.595	0.49	0.565	0.611
Ce_{SN} anomaly	0.640	0.455	0.777	0.634	0.419
Eu_{SN} anomaly	0.673	0.522	0.912	0.709	0.792

Supplementary table S2 continued.

sample name	Kalixälven	Luleälven	Råneälven	Piteälven	Skellefteälven
sampling date	15.05.2019	17.05.2019	14.05.2019	17.05.2019	20.05.2019
latitude	66.002494	65.826372	65.939371	65.741764	64.962231
longitude	22.852077	21.625377	22.133466	20.930794	19.888325
flow rate (m^3/s)	862	673	181	433	187
pH	6.31	6.28	6.04	6.55	6.44
conductivity (µS/cm)	30.0	24.0	20.0	18.0	29.0
temperature (°C)	6.40	5.90	4.10	7.90	9.20
DOC (mg/l)	10.3	5.81	8.71	6.96	7.01
µmol/l					
Ca	72.4	63.9	45.7	49.7	95.3
Fe	7.56	12.0	8.49	3.06	3.22
Na	50.0	49.5	41.9	41.6	49.2
Si	89.4	93.5	95.4	87.2	78.7
nmol/l					
Rb	19.6	15.3	19.1	13.6	12
Sr	120	130	121	111	160
Cs	0.0326	0.028	0.0616	0.0381	0.0436
Ba	39.5	41.2	39.2	29.0	54.1
U	0.791	0.398	0.233	0.805	0.271
pmol/l					
Sc	419	393	451	508	374
Y	1680	2930	2490	4570	1400
La	1370	2340	1340	3130	955
Ce	2080	3530	2250	3350	882
Pr	319	538	305	697	214
Nd	1200	1910	1110	2670	827
Sm	210	364	205	483	149
Eu	33.6	45.7	35.5	48.5	20.4
Gd	165	308	183	431	133
Tb	22.4	42.1	25.1	57.6	17.8
Dy	134	234	161	341	111
Ho	27.9	48.1	33.1	71.3	23.4
Er	81.0	146	105	201	73.6
Tm*	11.7	20.5	15.3	29.7	10.8
Yb	76.9	131	102	200	71.6
Lu	11.4	20.3	16.5	30.3	11.9
Tm recovery (%)	94.8	86.9	95.1	92.6	101
Sum REY (nmol/l)	7.42	12.6	8.38	16.3	4.90
Y/Ho	32.4	32.8	40.6	34.5	32.3
La_{SN}/Yb_{SN}	1.14	1.04	0.993	1.01	1.00
La_{SN}/Tb_{SN}	0.924	1.02	0.785	0.912	0.788
Tb_{SN}/Yb_{SN}	1.05	1.06	0.780	0.923	0.788
Ce_{SN} anomaly	0.743	0.863	0.913	0.557	0.470
Eu_{SN} anomaly	0.821	0.62	0.835	0.482	0.658

Supplementary table S2 continued.

sample name	Umeälven	Vindelälven	Västerdalälven downstream Fuluälven	Fuluälven	Ljöran
sampling date	20.05.2019	20.05.2019	31.07.2022	31.07.2022	31.07.2022
latitude	64.224782	64.576419	61.090088	61.308012	61.270121
longitude	19.357103	19.291091	13.316741	13.071528	12.848306
flow rate (m^3/s)	177	198	-	-	-
pH	6.58	6.95	7.22	7.67	7.53
conductivity (µS/cm)	27.0	31.0	24.6	32.9	16.3
temperature (°C)	11.8	11.8	17.1	18.3	15.1
DOC (mg/l)	7.92	5.50	2.71	3.43	3.10
µmol/l					
Ca	80.8	103	83.7	127	55.4
Fe	2.10	1.78	0.887	1.42	0.698
Na	46.5	38.1	43.2	54.6	34.7
Si	77.6	66.6	91.9	95.7	92.8
nmol/l					
Rb	14.5	13.4	9.11	7.1	7.94
Sr	145	222	172	244	111
Cs	0.068	0.0399	0.139	0.155	0.106
Ba	26.4	51.4	63.6	75.9	51.4
U	0.505	0.590	-	-	-
pmol/l					
Sc	542	390	262	189	390
Y	2190	2110	480	369	914
La	1910	1770	123	104	244
Ce	2180	1840	138	130	248
Pr	419	449	32.5	29.0	61.9
Nd	1540	1640	148	127	284
Sm	275	295	34.0	28.8	66.8
Eu	32.9	38.8	7.5	6.13	14.6
Gd	223	236	37.7	30.5	73.9
Tb	30.7	31.2	5.86	4.78	11.3
Dy	177	185	37.4	31.6	74.2
Ho	35.1	35.6	8.80	6.95	17.2
Er	106	106	28.2	22.6	55.9
Tm*	15.5	15.4	4.35	3.49	8.40
Yb	103	102	30.6	24.6	57.4
Lu	16.3	16.0	4.95	3.85	9.21
Tm recovery (%)	92.0	90.9	104	103	99.4
Sum REY (nmol/l)	9.25	8.87	1.12	0.921	2.14
Y/Ho	33.5	31.9	29.4	28.6	28.6
La_{SN}/Yb_{SN}	1.16	1.05	0.391	0.404	0.403
La_{SN}/Tb_{SN}	0.945	0.970	0.608	0.617	0.623
Tb_{SN}/Yb_{SN}	1.09	1.02	0.238	0.249	0.251
Ce_{SN} anomaly	0.622	0.491	0.524	0.568	0.502
Eu_{SN} anomaly	0.604	0.668	0.952	0.940	0.943

Supplementary table S2 continued.

sample name	Vanån	Västerdalälven downstream Vanån				
sampling date	01.08.2022	01.08.2022				
latitude	60.518574	60.525076				
longitude	14.215	15.023359				
flow rate (m^3/s)	6.70	47.9				
pH	6.70	6.75				
conductivity (µS/cm)	20.9	26.0				
temperature (°C)	18.0	19.3				
DOC (mg/l)	9.79	8.73				
µmol/l			LOQ 2019	LOQ 2022	SLRS-6 (n=2)	
Ca	71.9	78.6	17.4	82.9	216	
Fe	4.37	4.85	0.328	0.571	1.61	
Na	43.5	52.7	9.74	6.02	117	
Si	98.4	96.7	4.52	8.82	83	
nmol/l					SLRS-6 (n=4)	
Rb	8.30	9.59	10.1	0.158	19.2	
Sr	115	133	0.185	0.029	532	
Cs	0.153	0.109	0.0239	0.038	0.0322	
Ba	52.2	55.6	0.179	0.03	108	
U	-	-	0.0589	-	0.308	
pmol/l					SLRS-6 (n=4)	SLRS-6 preconcentrated (n=2)
Sc	942	943	11.9	27.7		397
Y	3570	3490	0.263	0.244	1550	1535
La	1340	1430	0.205	0.26	1860	1774
Ce	1780	2000	0.168	0.504	2230	2191
Pr	387	395	0.148	0.228	449	439
Nd	1580	1550	0.147	1.93	1690	1619
Sm	326	292	0.14	1.52	275	267
Eu	56.6	43.4	0.096	0.418	42.9	42.3
Gd	305	280	0.136	0.904	208	199
Tb	42.7	39	0.0891	0.221	23.1	23.9
Dy	258	236	0.117	0.97	144	132
Ho	56.4	53.0	0.0681	0.199	25.3	25.4
Er	170	168	0.0705	0.776	75.8	70.9
Tm*	25.4	25.3	0.0614	0.2	10.1	9.67
Yb	173	174	0.0537	0.943	68.0	65.8
Lu	29.1	29.6	0.0435	0.201	9.29	9.32
Tm recovery (%)	110	107				
Sum REY (nmol/l)	10.1	10.2				
Y/Ho	34.2	35.5				
La_{SN}/Yb_{SN}	0.583	0.685				
La_{SN}/Tb_{SN}	0.785	0.712				
Tb_{SN}/Yb_{SN}	0.458	0.488				
Ce_{SN} anomaly	0.573	0.591				
Eu_{SN} anomaly	0.815	0.69				

Supplementary table S3 REY and other trace element data for Swedish rivers sampled in 2014. The Ce_{SN} anomaly calculations are based on eq. 1, the Eu_{SN} anomaly calculations are based on eq. 2 and Tm* was calculated following eq. 3a and 3b. The flow rate data was taken from Swedish Meteorological and Hydrological Institute (2023)[1].

sample name<br>sampling year	Umeälven<br>early August 2014	Skellefteälven<br>early August 2014	Kalixälven<br>early August 2014	Dalälven<br>early August 2014	Västerdalälven<br>early August 2014
flow rate (m^3/s)	174-374	45.9-147	346-428	161-267	54.1-97.0
nmol/l					
Rb	14.7	13.5	15.5	15.2	6.68
Sr	236	204	292	156	133
Ba	32.7	54.0	54.4	63.8	62.3
U	0.194	0.181	0.57	0.66	<LOQ
pmol/l					
Y	273	469	440	2700	3070
La	183	282	349	1030	1000
Ce	113	265	391	1060	1640
Pr	45.2	68.4	84.4	275	297
Nd	181	259	320	1090	1230
Sm	34.7	49	58.6	217	256
Eu	5.14	6.77	9.88	36.4	52.8
Gd	34.5	50.3	52.6	216	267
Tb	4.76	7.36	7.11	35.3	37.4
Dy	27.2	42.6	43.3	184	235
Ho	5.44	8.67	8.81	43.8	50.8
Er	16.4	26.4	26.5	132	153
Tm*	2.41	3.78	3.89	19	21.6
Yb	16.2	24.7	26.0	125	139
Lu	2.57	3.79	3.93	24.6	20.4
SumREY (nmol/l)	0.945	1.57	1.82	7.19	8.47
Y/Ho	27.1	29.2	26.9	33.2	32.6
La_{SN}/Yb_{SN}	0.668	0.673	0.792	0.489	0.426
La_{SN}/Tb_{SN}	0.718	0.713	0.913	0.543	0.498
Tb_{SN}/Yb_{SN}	0.931	0.944	0.867	0.899	0.855
Ce_{SN} anomaly	0.292	0.472	0.547	0.479	0.670
Eu_{SN} anomaly	0.675	0.62	0.808	0.765	0.919

Supplementary table S4 REY data for Swedish lakes and rivers sampled in 1996. The Ce_{SN} anomaly calculations are based on eq. 1 and Eu_{SN} anomaly calculations are based on eq. 2. The flow rate data was taken from Swedish Meteorological and Hydrological Institute (2023)[1].

sample name<br>sampling year	Höljan<br>11.09.1996	Tåsan<br>11.09.1996	Likan<br>11.09.1996	Femtån<br>11.09.1996	Klarälven<br>10.09.1996	Övre Gla<br>09.09.1996	Österdalälven<br>15.09.1996	Rottnen<br>15.09.1996	Unnan<br>15.09.1996	Ämån<br>15.09.1996	Västerdalälven<br>10.09.1996
flow rate (m^3/s)	-	-	-	-	144	-	68	-	-	-	109
pmol/l											
Y	4970	6790	8310	6820	2310	1480	2600	2640	2800	3250	1770
La	3730	4960	6740	4610	1460	1070	1620	1570	1570	1830	575
Ce	6940	7230	10200	7500	1630	1250	2170	1390	2240	2880	792
Pr	962	1300	1750	1230	377	264	473	423	430	514	180
Nd	3390	4540	6050	4410	1390	952	1710	1520	1520	1860	740
Sm	566	778	986	752	237	166	314	270	275	343	156
Eu	81.9	87.0	119	82.1	34.7	26.1	32.9	27.4	34.3	44.5	32.3
Gd	480	656	840	647	217	146	278	236	242	302	162
Tb	69.4	91.3	113	90.2	30	19.1	36.1	33.1	35.0	42.7	23.1
Dy	407	551	668	526	180	108	209	199	217	262	142
Ho	87.5	119	141	116	38.2	24.3	43.6	43.3	47.2	56.6	30.9
Er	265	365	437	349	120	74.1	130	138	149	178	93.0
Tm	39.5	55.3	65.4	51.6	17.9	10.6	19.1	21.0	23.1	26.7	13.6
Yb	269	381	446	348	121	69.5	128	145	163	182	90.0
Lu	44.5	61.9	72.7	58.2	20.3	11.6	20.7	24.0	26.4	29.9	14.8
SumREY (nmol/l)	22.3	28	36.9	27.6	8.18	5.67	9.78	8.68	9.77	11.8	4.81
Y/Ho	30.6	30.8	31.8	31.7	32.6	32.8	32.1	32.9	32	30.9	30.9
La_{SN}/Yb_{SN}	0.821	0.768	0.893	0.783	0.711	0.905	0.749	0.638	0.570	0.595	0.377
La_{SN}/Tb_{SN}	1.00	1.01	1.11	0.952	0.904	1.04	0.835	0.882	0.837	0.800	0.464
Tb_{SN}/Yb_{SN}	0.820	0.76	0.804	0.822	0.786	0.872	0.897	0.724	0.681	0.744	0.814
Ce_{SN} anomaly	0.836	0.666	0.670	0.710	0.491	0.562	0.570	0.397	0.649	0.698	0.552
Eu_{SN} anomaly	0.714	0.553	0.594	0.535	0.695	0.762	0.506	0.493	0.604	0.628	0.923

Supplementary table S5 REY data for Swedish lakes and rivers sampled in 1994. The Ce_{SN} anomaly calculations are based on eq. 1 and Eu_{SN} anomaly calculations are based on eq. 2. The flow rate data was taken from Swedish Meteorological and Hydrological Institute (2023)[1].

sample name<br>sampling year	Glasfjorden<br>June 1994	Västerdalälven<br>June 1994	Klarälven<br>June 1994	Rottnan<br>June 1994	Kymmen<br>June 1994	Fryken<br>June 1994	Rottnen<br>June 1994	Gunnern<br>June 1994	Små Värmeln<br>June 1994	Stor Värmeln<br>June 1994
flow rate (m^3/s)	-	47-149	120-229	-	-	-	-	-	-	-
pmol/l										
Y	4650	3190	3940	7050	7640	6330	7230	7320	3170	2990
La	3260	1050	2780	6100	6860	5120	6190	5930	2030	1930
Ce	3870	1690	3780	7280	10600	7010	8600	8920	2120	2000
Pr	813	313	654	1440	1630	1240	1480	1440	508	472
Nd	3100	1330	2440	5230	6000	4660	5440	5240	2000	1840
Sm	557	278	433	888	1040	788	951	895	359	325
Eu	80.9	57.7	62.5	114	130	115	117	131	52.8	49.6
Gd	454	268	368	719	779	630	719	744	291	275
Tb	61.1	39.2	53.1	99.1	107	86.2	97.5	100	37.7	34.9
Dy	364	245	302	559	618	476	559	579	225	211
Ho	76.7	50.5	62.7	115	129	99.7	116	117	48.0	45.3
Er	230	153	188	342	382	301	344	357	149	143
Tm	32.1	21.3	25.7	48.1	51.9	41.4	48.5	48.5	20.2	20.1
Yb	212	135	170	311	331	285	314	320	142	135
Lu	spike	spike	spike	spike	spike	spike	spike	spike	spike	spike
SumREY (nmol/l)	17.8	8.82	15.3	30.3	36.3	27.2	32.2	32.1	11.2	10.5
Y/Ho	32.6	33.2	33.8	32.9	32.1	34.5	34.1	34	35.5	35.5
La_{SN}/Yb_{SN}	0.908	0.458	0.967	1.16	1.23	1.06	1.17	1.09	0.844	0.845
La_{SN}/Tb_{SN}	0.994	0.497	0.974	1.15	1.2	1.11	1.18	1.10	1.00	1.03
Tb_{SN}/Yb_{SN}	0.914	0.92	0.992	1.01	1.02	0.960	0.986	0.994	0.843	0.821
Ce_{SN} anomaly	0.547	0.651	0.674	0.577	0.745	0.622	0.677	0.709	0.465	0.468
Eu_{SN} anomaly	0.732	0.961	0.712	0.649	0.657	0.742	0.644	0.727	0.742	0.753

Supplementary table S6 DOC, Yb and Sc data for rivers and lakes in Germany and France used for comparison with the data from Swedish rivers.

	Lake Bullen	Achter	Murg	Ill	Rhine River
Sc (pmol/l)	152	128	414	134	68.1
Yb (pmol/l)	7.82	31.3	83.7	24.1	13.5
DOC (mg/l)	5.33	1.95	3.64	1.55	2.20

Supplementary table S7 Sc/Yb ratios for the suspended particles for the samples from 2019 and 2022

	Sc/Yb
Råneälven	1.97
Kalixälven	3.40
Piteälven	1.58
Luleälven	3.01
Skellefteälven	1.29
Umeälven	1.69
Indalsälven	3.37
Dalälven	1.85
Västerdalälven	1.90
Österdalälven	1.08
Klarälven	1.57
Västerdalälven downstream Fuluälven	2.48
Fuluälven	2.07
Ljöran	2.10
Vanån	1.06
Västerdalälven downstream Vanån	1.39

Supplementary table S8 Comparison of ICP-MS measurements of Sc with and without using KED mode

sample name	Sc (pmol/l) without KED	Sc (pmol/l) with KED	relative standard deviation (%)
Vindelälven	420	385	4.33
SLRS-6	359	360	0.14

Supplementary table S9 Comparison between the two different preconcentration methods: Preconcentration with and C18 cartridges loaded with ethyl-hexyl phosphates (method 1) and preconcentration with the Nobias PA-1 resin (method 2).

pmol/l	Piteälven method 1	Piteälven method 2	rsd (%)	Kalixälven method 1	Kalixälven method 2	rsd (%)
Y	4370	4570	4.38	1770	1680	5.51
La	3020	3130	3.65	1430	1370	4.62
Ce	3580	3350	6.83	2270	2080	8.99
Pr	755	697	8.30	330	319	3.39
Nd	2690	2670	0.998	1220	1200	2.11
Sm	511	483	5.67	203	210	3.13
Eu	49	48.5	1.14	34.6	33.6	2.89
Gd	426	431	1.23	172	165	3.84
Tb	60.7	57.6	5.30	24.1	22.4	7.30
Dy	344	341	0.838	135	134	0.511
Ho	69.6	71.3	2.45	28.7	27.9	2.93
Er	216	201	7.71	83.8	81.0	3.50
Tm*	-	-	-	-	-	-
Yb	201	200	0.0917	79.1	76.9	2.83
Lu	28.9	30.3	4.58	11.6	11.4	1.09

Supplementary table S10 Calculations of the Sc and REY concentration in the Dalälven assuming that Österdalälven contributes 54% towards Dalälven.

	Dalälven Measured concentration	Dalälven Calculated concentration	Deviation of measured and calculated values
pmol/kg			
Sc	1072	905	18 %
Y	5354	4970	8%
La	2115	1987	6%
Ce	2716	2370	15%
Pr	539	514	5%
Nd	2135	2028	5%
Sm	434	416	4%
Eu	65.0	61.1	6%
Gd	399	380	5%
Tb	54.5	53.5	2%
Dy	335	320	5%
Ho	74.9	70.4	6%
Er	223	212	5%
Tm*	32.9	31.5	5%
Yb	221	213	4%
Lu	33.3	34.4	3%

References

1. Swedish Meteorological and Hydrological Institute. SMHI Vattenweb. https://vattenwebb.smhi.se/station/ (2023).
